# Role of bronchoscopy during non-invasive ventilation in hypercapnic respiratory failure

**DOI:** 10.1186/cc12074

**Published:** 2013-03-19

**Authors:** W Rady, A Abouelela, A Aly, W Youssef

**Affiliations:** 1 Alexandria University, Alexandria, Egypt

## Introduction

Non-invasive positive-pressure ventilation (NIPPV) is the first-line treatment for hypercapnic acute respiratory failure (ARF) secondary to COPD exacerbation in selected patients. Limited data exist supporting the use of fiberoptic bronchoscopy (FOB) during this clinical setting. The aim of this study is to assess the role of FOB during NIPPV in patients with decompensated COPD acute exacerbation.

## Methods

This study is a randomized prospective case-control pilot study carried out on 50 patients admitted to critical care units at Alexandria University Hospital, Egypt suffering from hypercapnic ARF secondary to COPD exacerbation with Kelly Matthay Score from 2 to 4. All patients received NIPPV. Patients were divided randomly into two equal groups: group I (cases, 25 patients) were subjected to additional intervention, early FOB during the first 6 to 12 hours from admission; while group II (control, 25 patients) received the conventional treatment and NIPPV only. Outcome parameters measured were changes in ABG data, duration of NIPPV, rate of its success, ICU stay and mortality as well as the safety of FOB and possible complications.

## Results

No significant difference was detected between the two groups regarding the baseline characteristics. No serious complications happened from FOB, oxygen desaturation occurred in 4/25 patients (16%), tachycardia in 2/25 patients (8%). In group I, 23 patients (92%) were successfully weaned from NIPPV versus 16 patients (64%) in group II (*P *= 0.037). Total duration of NIPPV was 28.52 hours in group I versus 56.25 hours in group II (*P *= 0.001). Length of ICU stay was 4.84 days in group I versus 8.68 days in group II (*P *= 0.001). Only one patient died in group I versus three patients in group II (*P *= 0.609).

## Conclusion

The early application of FOB during NIPPV in patients with ARF due to COPD exacerbation was shown to be safe. Significant improvement in the outcome of patients who underwent FOB was noticed in terms of improved ABG data, shorter duration of NIPPV, higher percentage of success and shorter ICU stay while no significant difference was detected in mortality.

## References

[B1] Scala R Crit Care 2010 14 R80 10.1186/cc8993 20429929 PMC2887203

[B2] Ambrosino N Eur Respir J 2008 31 874 886 10.1183/09031936.00143507 18378782

[B3] Heunks LM Intensive Care Med 2010 36 143 147 10.1007/s00134-009-1662-6 19774365 PMC2807591

